# Oral Health and Nutritional Characteristics of Adults With Morbid Obesity: A Multivariate Analysis

**DOI:** 10.3389/fnut.2020.589510

**Published:** 2020-11-20

**Authors:** Maria Carolina Salomé Marquezin, Samuel de Carvalho Chaves-Júnior, Irineu Rasera, Elisane Rossin Pessotti Pacheco, Maria Beatriz Duarte Gavião, Elsa Lamy, Paula Midori Castelo

**Affiliations:** ^1^Department of Pharmaceutical Sciences, Institute of Environmental, Chemical and Pharmaceutical Sciences, Universidade Federal de São Paulo (UNIFESP), São Paulo, Brazil; ^2^Department of Pediatric Dentistry, Piracicaba Dental School, University of Campinas (UNICAMP), São Paulo, Brazil; ^3^Piracicaba Medical School, Anhembi Morumbi University, São Paulo, Brazil; ^4^Department of Physiotherapy, Methodist University of Piracicaba (UNIMEP), Piracicaba, Brazil; ^5^MED Mediterranean Institute for Agriculture, Environment and Development, University of Évora, Évora, Portugal

**Keywords:** morbid obesity, oral health, comorbidities, saliva, nutrition

## Abstract

The relationship between oral health and nutritional aspects are complex, especially in individuals with chronic diseases and comorbidities, such as morbid obesity. Thus, the aim of the present study was to identify oral health and nutritional-related patterns in 113 individuals, aged 19–68 years (92 females), seeking treatment for morbid obesity. Sociodemographic variables and medical records were examined, in addition to the consumption of fruit, vegetables, candies, and processed foods. Measures of body mass index, neck, waist and hip, caries experience (DMFT index), Community Periodontal Index (CPI index), and salivary physicochemical aspects were gathered. Aspects of oral health-related quality of life and symptoms of dry mouth were evaluated by means of Oral Health Impact Profile (OHIP-14) and Xerostomia Inventory-XI questionnaires. K-means cluster analysis and, subsequently, comparisons between clusters (one-way ANOVA) were performed (α = 5%). Three clusters were generated: Cluster 1 (labeled “Young”; *n* = 77) was characterized by younger participants with higher BMI, who reported the use of distractors while eating, the smallest number of meals/day, and who consumed sweetened drinks and processed food the day before. Cluster 2 (labeled “Diabetic individuals”; *n* = 12) was characterized by older participants with the highest proportion of diabetic participants (100% were diabetic; 73% insulin users), lower BMI, higher DMFT index and OHIP-14 and xerostomia scores, and who reported having consumed fruit and vegetables the day before. Finally, Cluster 3 (labeled “Poor periodontal health”; *n* = 24) was characterized by participants with the worse periodontal condition (higher CPI), and lower salivary flow, pH, and buffer capacity. Cluster 1 and 2 were the groups that showed higher demand for nutritional and dietetic counseling, because of the poor eating behavior and higher serum glucose levels, respectively. On the other hand, Cluster 2 and 3 showed the higher demand for oral rehabilitation and dental treatment because of the loss of teeth and worse periodontal condition, respectively, besides the need for dietetic counseling. This sample of individuals with morbid obesity showed very unique oral-health and nutritional characteristics and special needs patterns that should be identified to adjust or change unhealthy habits, thus improving the assistance of this condition.

## Introduction

Obesity is considered a multifactorial and chronic condition that results in excess fat storage due to biological, behavioral, and environmental factors (1). According to the World Health Organization (2018) (2), 13% of adults have obesity (BMI of 30 to <40 Kg/m^2^) in the world, which is associated with comorbidities such as type 2 diabetes mellitus, cardiovascular diseases, hyperlipidemia, sleep apnea syndrome, respiratory diseases, neoplasms, endocrine disorders, periodontal disease, psychosocial problems, among others (3). The prevalence of morbid obesity, characterized as BMI of 40 Kg/m^2^ or higher, has also increased over the last 30 years (4). In the United States, an increase in the percentage of adults with morbid obesity was observed from 5.7% in 2007 to 7.7% in 2016 (5). In Brazil, an upward trend in morbid obesity prevalence was also observed, with women showing higher rates (1.3% in 2006 and 1.9% in 2017) than men (0.9% in 2006 and 1.4% in 2017) (6).

A worse quality of life is observed in people with excess of weight, relative to physical and mental health impairments, in addition to social problems (7). Obesity and oral health may share similar causal and behavioral mechanisms (8), mainly related to the diet. Previous studies reported an association between obesity and several oral diseases, such as dental caries (9), periodontal disease, tooth loss (7, 10, 11), and xerostomia (defined as the subjective perception of oral dryness) (9, 12).

In the previously mentioned studies, authors observed a higher number of tooth losses and higher frequency of periodontal disease in individuals with obesity (10, 13), pointing out their greater demand in health services and nutritional counseling. Considering the morbid obesity, there is a lack of such information and, thus, it is essential to deepen the knowledge about oral health characteristics of these individuals and their special needs, in order to ensure the better assistance, especially in individuals with morbid obesity.

The hypothesis of the study was that even among individuals with similar anthropometric status and seeking treatment for morbid obesity, many oral and nutritional health-related aspects may vary, given the multifactorial nature of these aspects; thus, when designing health care therapies, it is crucial to understand the specific needs of each group. In this sense, the aim was to perform an exploratory study using cluster analysis to identify oral health and nutritional-related patterns in adults with morbid obesity.

## Materials and Methods

### Study Design

This is a cross-sectional study approved by the Ethical Research Committee of the Federal University of São Paulo (Protocol No. 1201/2017). All the individuals read and signed an informed consent form to take part in the study.

### Sample

The study included a convenience sample of 113 adults, aged between 19 and 68 years with morbid obesity, who were evaluated just before starting a dietary program prior to gastroplasty surgery in the Bariatric Clinic of Piracicaba (SP, Brazil), between the years 2018 and 2019. Of them, 92 were females.

The inclusion criteria were individuals with morbid obesity (BMI of 40 Kg/m^2^ or higher) of both sexes, with at least 20 natural teeth or who use dental prosthesis. The exclusion criteria were individuals presenting epilepsy, cancer, rheumatoid arthritis, bucco-dentofacial diseases or traumas, tobacco use, illicit drugs, Sjögren's syndrome, systemic lupus erythematosus, sarcoidosis, alcoholic beverage, and extensive tooth loss.

### Anamnesis and Interview

Data collection was performed by means of a structured questionnaire applied during an interview by one of the authors (MCSM). The following data were investigated: date of birth, age, declared ethnic group (black/white/mixed), marital status, educational level, family income, weight, height, use of chronic medications, and any diseases or health conditions (hypertension, diabetes mellitus, dependent insulin), in addition to dental history. Measures of fasting glucose, uric acid, calcium, high density lipoprotein (HDL), low density lipoproteins (LDL), and total cholesterol laboratory analysis were gathered from medical records.

The qualitative assessment of food consumption habits was performed using a brief questionnaire proposed by the Brazilian Ministry of Health (14), which identifies the eating behavior and the consumption of healthy foods like fruit, vegetables, meat and beans, and unhealthy foods such as sausages, artificial juices, soft drinks, instant noodles, cookies, snacks, and sweets (processed and ultra-processed food). Participants answered questions regarding the consumptions of these items in the day before interview (yes/no). Questions also covered the habit of watching TV, using the computer and/or cell phone during the meal and the number of meals per day (day to day).

The Brazilian-validated version of the Oral Health Impact Profile (OHIP-14) was applied during the interview (15) and provided a comprehensive measure of the self-reported dysfunction, discomfort and disability attributed to the oral condition. This consisted of 14 items divided in 7 domains (functional limitation, pain, psychological discomfort, physical disability, psychological disability, social disability, and handicap). For each OHIP-14 item, participants were asked how frequently they had experienced the impact of that item. Responses were made on a 5-point Likert scale: 0 “never,” 1 “hardly ever,” 2 “occasionally,” 3 “fairly often,” 4 “very often.” OHIP-14 total scores, ranging from 0 to 56 points, were obtained by summing the responses on all 14 questions (items). Higher scores imply poorer oral health-related quality of life and thus, lower satisfaction.

Xerostomia was measured using the Xerostomia Inventory XI. Items were scored on a five-point unidirectional rating scale that rated the frequency of experiencing dry mouth symptoms from “Never” to “Very often.” The scores range from 11 (no xerostomia) to a maximum of 55 (severe xerostomia) (16).

### Clinical Examination

Physical examination was carried out at the clinic by a trained examiner (MCSM) and included measures of body mass index (BMI kg/m^2^), neck, waist, and hip circumferences. Neck circumference was measured using a flexible ruler at the thyroid cartilage level (17). Waist circumference was measured to the nearest centimeter between the iliac crest and the lower rib (18) and hip circumference is measured at the height of the largest horizontal diameter. All measurements were performed twice and the mean was considered as the final value.

The oral examination was also performed at the clinic, in a private room, using a clinical mirror, probe and mouth retractors, according to the World Health Organization recommendations (19). Caries experience was evaluated by assessing the number of decayed, missing, and filled permanent teeth (DMFT). Periodontal status was assessed using the Community Periodontal Index (CPI), which has three indicators: gingival bleeding, calculus, and depth of the periodontal pockets; the possible scores are: 0- Healthy; 1- Bleeding after probing; 2- Calculus; 3- Pocket 4–5 mm; 4- Pocket 6 mm or more. The highest CPI score for each sextant was recorded from six teeth-indexes (16, 11, 26, 36, 31, and 46). Each tooth-index was carefully examined by a trained and calibrated examiner (SCCJ), using a mirror and WHO probe with a 0.5 mm ball tip (19) and applying a force of about 20 g (as recommended by the methodology during training).

### Evaluation of Salivary Parameters

Stimulated saliva (SS) was collected from participants chewing on 0.3 g of an inert and tasteless material (Parafilm, Merifeld, USA) for 5 min into a cooled tube, in the morning, with all of them having refrained from eating, drinking or brushing their teeth for a minimum of 2 h before collection.

Salivary pH was determined immediately after collection, using a portable pH meter (Orion 3 Star Benchtop, Thermo Electron Corporation, USA). After calibration, the electrode was immersed in a Falcon tube containing saliva for 30 s for measurement. Further, buffer capacity was measured according to the methodology described previously (20, 21).

### Taste Sensitivity

The evaluation of taste sensitivity was conducted using an adaptation of a validated methodology (22), called three-drop-method which uses four concentrations of each basic tastes (salty, sweet, sour, and bitter). In the present study, only the lowest concentration of each stimuli was used: salty–sodium chloride (0.016 g/mL), sweet–sucrose (0.05 g/mL), acid–citric acid (0.0125 g/mL), bitter–quinine hydrochloride (0.0001 g/mL), which were administered in a dropper (three drops) on the back of the tongue, with 1 drop of the tastant solution and 2 drops of distilled water, in order to verify the sensitivity to the lower concentrated tastants, which is near to the limit threshold.

The order of presentation of the tests was drawn for each individual. The participant should choose, for each of the tests, one of the four options: sweet, salty, bitter, or acid (sour), with no time limit for the test (forced choice). Between each test, individuals were instructed to rinse their mouths with mineral water to avoid residual taste that could confuse them. For each test correctly identified, the volunteer received 1 point, and the incorrect answers, either for not being able to identify the flavor or for having confused it with another flavor, do not add points (maximum of 4 points).

### Statistical Analysis

Statistical analysis was performed using SPSS 26.0 software considering an alpha level of 5% by an Applied Statistics Spec (PMC). Descriptive analysis consisted of means, standard deviation, and percentages.

Firstly, hierarchical cluster analysis using farthest neighbor method for calculating distances between clusters was performed to obtain the dendrogram and analyze the range of clusters for further running K-means analysis, which was performed to identify groups of participants with similar oral health and nutritional-related variables. After *Z*-score transformation, the analysis included the following variables: age, sex, BMI, diagnostic of diabetes, insulin therapy, salivary parameters, taste sensitivity, oral health parameters, OHIP-14 and xerostomia scores, and food consumption behavior aspects. The final number of clusters was based on the interpretability and reliability of the cluster solution, and the differences between clusters were assessed by *F*-test for clustering validation (it is important to mention that F tests should be interpreted only for descriptive purposes, as the clusters were chosen to maximize the differences between each case and the other clusters).

Further, One-way ANOVA and Bonferroni's post-test were used to compare the clinical characteristics and aspects of oral health-related quality of life (OHIP-14 scores) and xerostomia scores between clusters.

## Results

The study included 113 adults between the ages of 19 and 68 years with morbid obesity. K-means cluster analysis generated three reliable and meaningful clusters of individuals, varying significantly according to the oral health-nutritional-related aspects of the participants. Table 1 shows the final cluster centers (*Z*-scores cluster-variables means) which defined the three clusters gathered from multivariate analysis. According to the taxonomy description, Cluster 1 (labeled “Young”; *n* = 77) was characterized by younger participants with higher BMI, who reported to use distractors while eating, having the smallest number of meals/day, and who consumed sweetened drinks and processed food the day before examination.

**Table 1:** Final cluster centers (means of *Z*-scores) of the oral-nutritional-related variables (important differences which identify the clusters are colored).

	**Cluster 1 Young**	**Cluster 2 Diabetic individuals**	**Cluster 3 Poor periodontal health**	**F-test**
Number of cases	77	12	24	
Age	−0.3281	1.3656	0.3698	23.920
Sex	0.0435	−0.1642	−0.0576	0.271
Diabetes	−0.2785	1.9694	−0.0912	48.901
Insulin intake	−0.2761	2.5352	−0.2761	131.060
Body mass index	0.1083	−0.3607	−0.1626	1.559
Salivary flow	0.2487	−0.2736	−0.7696	10.555
Salivary pH	0.2091	−0.1282	−0.7303	8.024
Salivary buffer capacity	0.1406	0.1101	−0.5893	4.577
Taste sensitivity	0.0625	−0.0183	−0.1889	0.574
Periodontal condition	−0.2272	−0.1035	0.8366	11.633
DMFT index	−0.3029	1.0615	0.4669	15.342
OHIP-14	−0.1431	0.9911	0.0049	6.842
Xerostomia score	−0.0542	0.9314	−0.2530	6.180
Distractors use while eating	0.2824	−0.6426	−0.5889	10.878
Number of meals	−0.2965	0.6162	0.6861	12.946
Fruit consumption	−0.1827	0.6606	0.27588	4.908
Vegetable consumption	−0.0677	0.6615	−0.0584	2.470
Sweetened drink consumption	0.1577	−0.2222	−0.40425	3.324
Candy consumption	0.0704	−0.2964	−0.0901	0.768
Processed/ultra-processed food consumption	0.1488	−0.5036	−0.2574	3.126

*DMFT, decayed, missing, and filled teeth; OHIP-14, Oral Health Impact Profile*.

Cluster 2 (labeled “Diabetic individuals”; *n* = 12) was characterized by older participants with the highest proportion of diabetic subjects (100%; 73% insulin users), lower BMI, higher DMFT index, OHIP-14, and xerostomia scores, and who reported having consumed fruit and vegetables the day before. Finally, Cluster 3 (labeled “Poor periodontal health”; *n* = 24) was characterized by participants with the worse periodontal condition (higher CPI), and lower salivary flow, pH and buffer capacity. On the other hand, the sensitivity to the lower concentrations of sweet, salt, acid, and bitter tastes did not significantly contribute for clustering individuals.

Table 2 shows the characteristics of the clusters according to sociodemographic and clinical aspects. Sociodemographic aspects such as schooling (>8 years) and percentage of individuals who reported as ethnic group “white” were similar between groups. Among the measures of fasting serum glucose, uric acid, calcium, HDL, LDL, and total cholesterol, only fasting serum glucose level was different between clusters (*p* < 0.001; *eta* partial^2^ = 0.22; power = 0.99), with a mean higher than 150 mg/dL in the cluster 2 labeled “Diabetic individuals.” This cluster also showed the higher DMFT index; a closer look at the DMFT index revealed that the component “missing” contributed most to this difference (means: 1.9, 10.8, and 5.2 for Cluster 1, 2 and 3, respectively).

**Table 2:** Description of the demographic and clinical characteristics of the clusters.

		**Cluster 1Young**	**Cluster 2 Diabetic individuals**	**Cluster 3Poor periodontal health**
	Number of cases	77	12	24
Age	Mean (±SD)	34.4 (8.8)	50.8 (8.2)	41.9 (8.7)
Sex (females)	%	83	75	79
Declared ethnic group (black/white/mixed)	%	17/52/31	18/55/27	13/58/29
Marital status (married or cohabiting couple)	%	51	58	74
Schooling (>8y)	%	97	100	96
Income (number of min wages)	Mean (±SD)	2.3 (1.7)	1.9 (1.1)	3.1 (2.6)
Body mass index (Kg/m^2^)	Mean (±SD)	47.8 (9.1)	41.1 (2.1)	45.6 (8.0)
Neck circumference (cm)	Mean (±SD)	40.0 (4.0)	41.2 (4.3)	39.0 (3.9)
Waist-hip ratio (cm)	Mean (±SD)	0.6 (0.2)	0.6 (0.2)	0.6 (0.2)
Diabetics / Insulin users	%	9 / 0	100 / 73	17 / 0
Number of meals/day	Mean (±SD)	3.1^A^ (1.0)	4.0^B^ (1.2)	4.2^B^ (0.9)
Fasting glucose (mg/dL)	Mean (±SD)	101.8 ^A^ (19.4)	157.7^B^ (51.7)	128.2^A^ (58.5)
Uric acid (mg/dL)	Mean (±SD)	5.8 (1.1)	5.6 (1.4)	5.6 (1.8)
Calcium (mg/dL)	Mean (±SD)	9.4 (0.4)	9.5 (0.4)	9.4 (0.4)
High density lipoprotein (mg/dL)	Mean (±SD)	44.1 (11.2)	41.8 (3.5)	49.0 (11.2)
Low density lipoproteins (mg/dL)	Mean (±SD)	103.5 (30.8)	102.7 (38.8)	107.6 (37.7)
Total cholesterol (mg/dL)	Mean (±SD)	176.8 (33.3)	181.2 (42.0)	182.5 (41.5)
Stimulated salivary flow (mL/min)	Mean (±SD)	1.2^A^ (0.6)	0.9^AB^ (0.6)	0.7^B^ (0.4)
Stimulated salivary pH	Mean (±SD)	7.4^A^ (0.4)	7.2^AB^ (0.4)	7.0^B^ (0.6)
Buffer Capacity	Mean (±SD)	4.7^A^ (1.1)	4.6^AB^ (1.3)	3.9^B^ (0.6)
Community Periodontal Index	Mean (±SD)	2.1^A^ (0.6)	2.2^A^ (0.7)	2.7^B^ (0.9)
DMFT index	Mean (±SD)	8.3^A^ (6.1)	17.6^B^ (6.0)	12.3^B^ (4.7)
Component D (decayed)	Mean (±SD)	0.5 (0.8)	0.5 (0.7)	0.7 (0.9)
Component M (missed)	Mean (±SD)	1.9^A^ (2.7)	10.8^B^ (7.7)	5.2^C^ (6.8)
Component F (filled)	Mean (±SD)	5.8 (4.9)	6.1 (5.0)	7.5 (4.8)

A ≠ B in the same column (p < 0.05; One-way ANOVA; Bonferroni's post-test).

*DMFT, decayed, missing, and filled teeth*.

As mentioned above, Cluster 3 labeled “Poor periodontal health” also showed the most severe periodontal disease (on average, this cluster achieved a maximum grade of 2.7 ± 0.9), the higher mean number of decayed and filled teeth and the lowest means of salivary flow, pH, and buffer capacity (Table 2).

**Figure 1:**
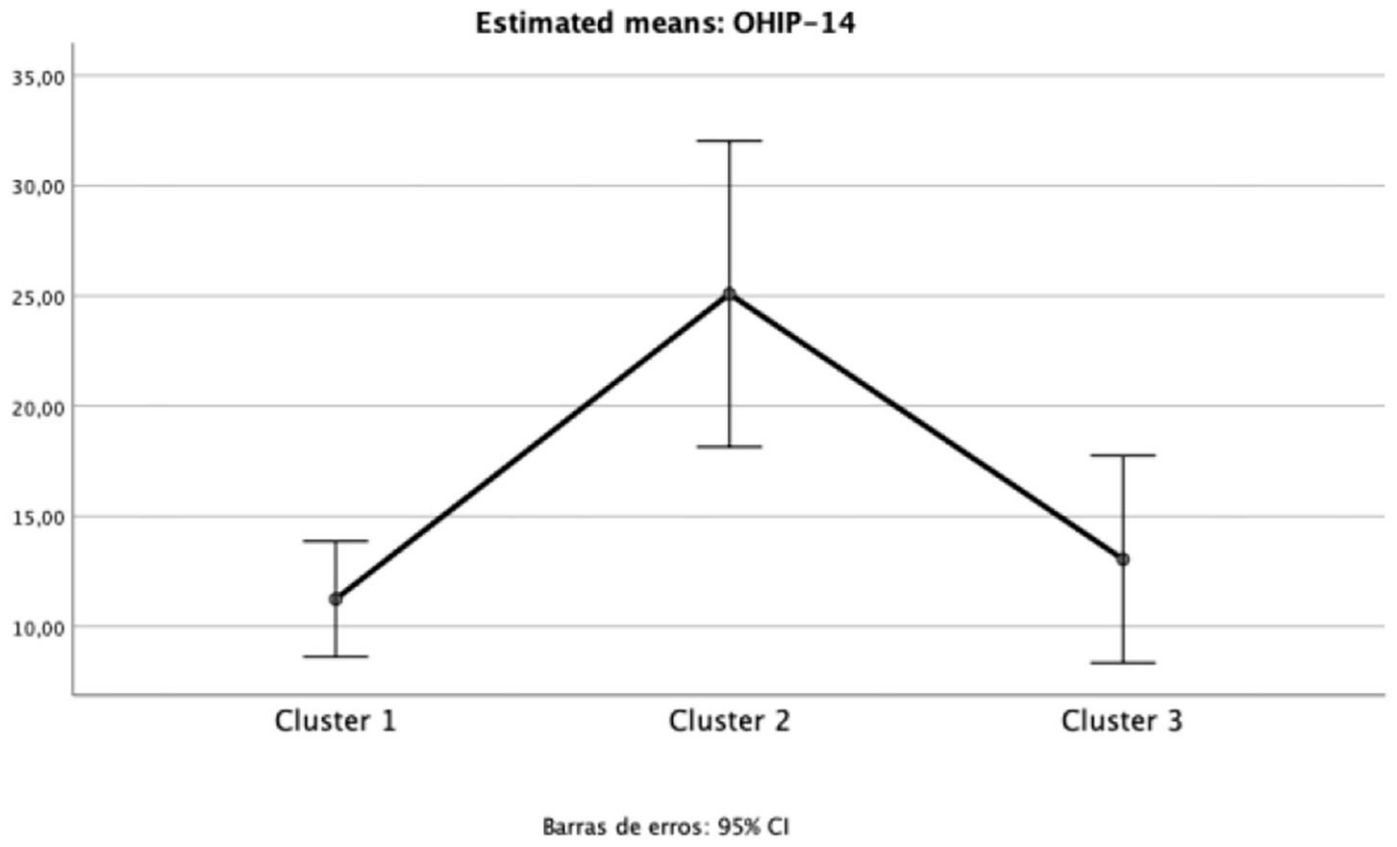
Mean and 95%CI of Oral Health Impact Profile (OHIP-14) total scores for each cluster (one-way ANOVA *p* < 0.05; *eta* partial squared = 0.11; power = 92%).

**Figure 2:**
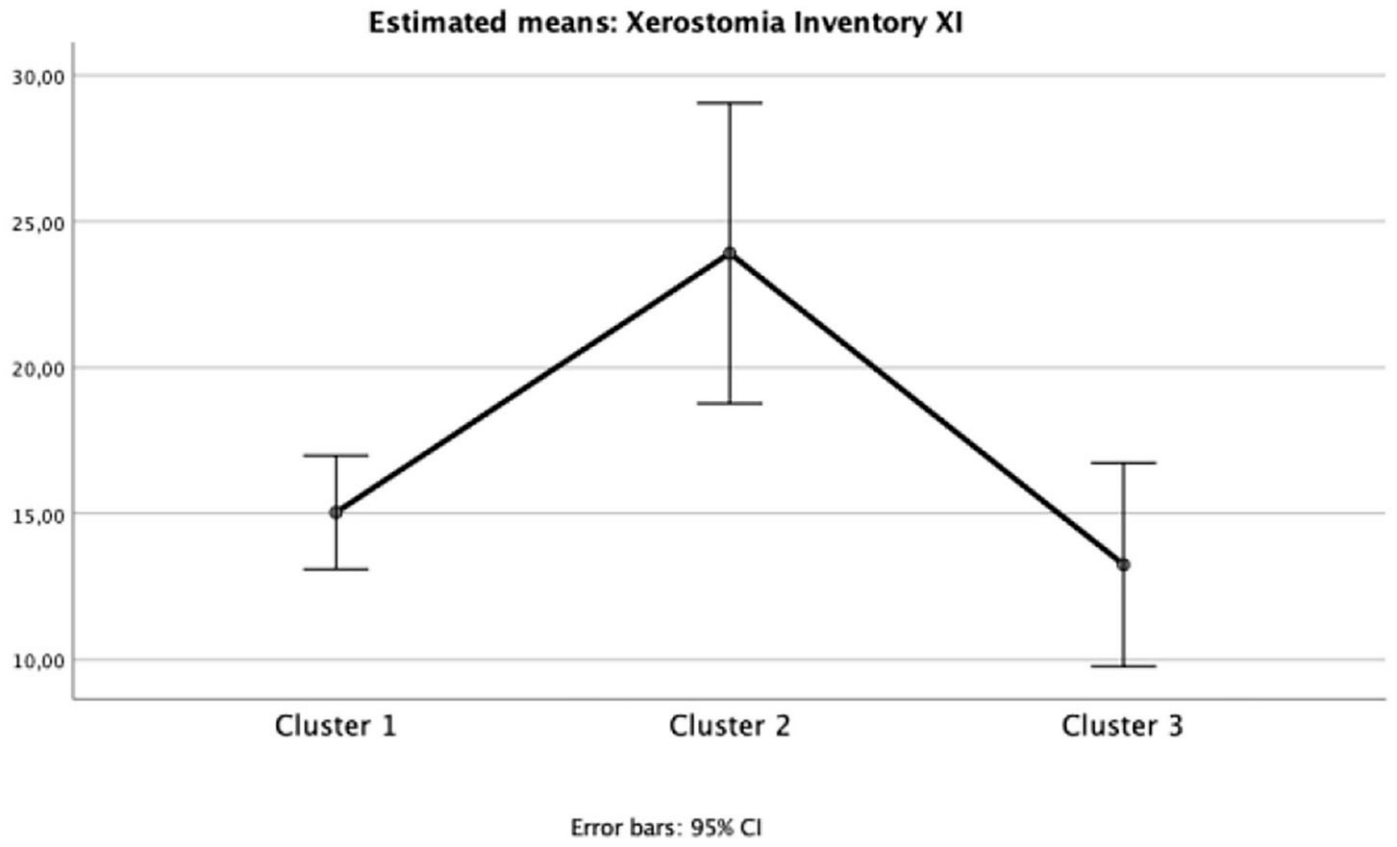
Mean and 95%CI of Xerostomia Inventory XI total scores for each cluster (one-way ANOVA *p* < 0.05; *eta* partial squared = 0.10; power = 88%).

The impact of oral health on quality of life was evident in Cluster 2 (“Diabetic individuals”) (Table 3), probably because of the large number of missing teeth as shown in Table 2. Besides, symptoms of xerostomia were also worse in individuals of Cluster 2; OHIP-14 and Xerostomia inventory XI total score of Cluster 2 were significantly higher than that found in the other clusters (mean 25.1 ± 9.5, *p* = 0.002, *eta* partial^2^ = 0.10; mean 23.9 ± 5.9, *p* = 0.003, *eta* partial^2^ = 0.11, power >80%, respectively) (Figures 1, 2). The largest differences were perceived in the need to interrupt meals because of the oral conditions (OHIP-14, question 9) and difficulties when swallowing certain foods (Xerostomia inventory, question 7), emphasizing the perception of discomfort and difficulties that these individuals encounter during meals.

**Table 3:** Oral health-related quality of life and xerostomia scores description according to clustering groups (mean and SD).

		**Cluster 1 Young**	**Cluster 2 Diabetic individuals**	**Cluster 3 Poor periodontal health**	***p*-value (power)**
**OHIP-14**	Have you had trouble pronouncing any words because of problems with your teeth, mouth or dentures?	0.4 (0.9)	1.1 (0.9)	0.6 (1.2)	0.154 (0.40)
	Have you felt that your sense of taste has worsened because of problems with your teeth, mouth or dentures?	0.5^A^ (0.9)	1.4^B^ (1.5)	0.6^AB^ (1.1)	0.029 (0.66)
	Have you had painful aching in your mouth?	1.3 (1.1)	2.1 (0.8)	1.9 (1.1)	0.025 (0.69)
	Have you found it uncomfortable to eat any foods because of problems with your teeth, mouth or dentures?	1.3 (1.2)	2.2 (1.5)	1.3 (1.3)	0.088 (0.49)
	Have you been self-conscious because of your teeth, mouth or dentures?	1.7 (1.4)	2.7 (1.4)	1.9 (1.5)	0.106 (0.46)
	Have you felt tense because of problems with your teeth, mouth or dentures?	1.1^A^ (1.5)	2.3^B^ (1.6)	1.0^AB^ (1.2)	0.031 (0.65)
	Has your diet been unsatisfactory because of problems with your teeth, mouth or dentures?	0.7^A^ (1.2)	2.0^B^ (1.8)	1.1^AB^ (1.4)	0.011 (0.78)
	**Have you had to interrupt meals because of problems with your teeth, mouth or dentures?**	**0.5**^**A**^ **(0.9)**	**1.8**^**B**^ **(0.9)**	**0.5**^**A**^ **(0.8)**	**<0.001 (0.99)**
	Have you found it difficult to relax because of problems with your teeth, mouth or dentures?	0.8 (1.2)	1.7 (0.9)	0.8 (1.0)	0.058 (0.56)
	Have you been a bit embarrassed because of problems with your teeth, mouth or dentures?	1.1^A^ (1.5)	2.3^B^ (1.6)	1.4^AB^ (1.8)	0.049 (0.59)
	Have you been a bit irritable with other people because of problems with your teeth, mouth or dentures?	0.7 (1.3)	1.3 (1.0)	0.5 (1.1)	0.198 (0.34)
	Have you had difficulty doing your usual jobs because of problems with your teeth, mouth or dentures?	0.4^A^ (0.8)	1.5^B^ (1.1)	0.4^A^ (1.1)	0.003 (0.88)
	Have you felt that life in general was less satisfying because of problems with your teeth, mouth or dentures?	0.6^A^ (1.1)	2.1^B^ (1.5)	0.9^A^ (1.5)	0.001 (0.94)
	Have you been totally unable to function because of problems with your teeth, mouth or dentures?	0.3 (0.7)	0.7 (1.3)	0.4 (0.9)	0.142 (0.40)
	OHIP-14 total score	11.2^A^ (11.1)	25.1^B^ (9.5)	13.0^A^ (12.0)	0.002 (0.92)
**Xerostomia Inventory-XI**	I sip liquids to aid in swallowing food	2.1 (1.5)	1.6 (1.2)	1.9 (1.6)	0.624 (0.13)
	My mouth feels dry when eating a meal	1.1^A^ (1.3)	2.4^B^ (1.7)	1.0^A^ (1.4)	0.013 (0.76)
	I get up at night to drink	2.0 (1.5)	2.7 (1.8)	1.8 (1.4)	0.344 (0.24)
	My mouth feels dry	1.9 (1.4)	2.5 (1.4)	2.0 (1.4)	0.486 (0.17)
	I have difficulty in eating dry foods	1.0 (1.3)	2.0 (1.7)	1.2 (1.5)	0.099 (0.47)
	I suck sweets or cough lollies to relieve dry mouth	0.9^AB^ (1.3)	1.9^A^ (1.6)	0.6^B^ (1.1)	0.025 (0.68)
	**I have difficulties swallowing certain foods**	**0.6**^**A**^ **(0.9)**	**1.9**^**B**^ **(1.7)**	**0.3**^**A**^ **(0.7)**	**<0.001 (0.97)**
	The skin of my face feels dry	0.99^A^ (1.4)	2.3^B^ (1.9)	0.8^A^ (1.4)	0.011 (0.78)
	My eyes feel dry	0.8 (1.2)	1.4 (1.9)	0.9 (1.4)	0.376 (0.22)
	My lips feel dry	1.9 (1.5)	3.0 (1.2)	1.7 (1.6)	0.048 (0.59)
	The inside of my nose feels dry	1.6^A^ (1.5)	3.0^B^ (1.4)	1.7^A^ (1.3)	0.003 (0.88)
	Xerostomia Inventory-XI total score	15.0^A^ (8.9)	23.9^B^ (5.9)	13.3^A^ (9.6)	0.003 (0.88)

*A ≠ B in the same column (p < 0.05; One-way ANOVA; Bonferroni's post-test); the parameters for which p < 0.001 were highlighted in bold*.

*BMI, body mass index; OHIP-14, Oral Health Impact Profile*.

## Discussion

The relationship between oral health and nutritional status are complex and multifactorial, especially in individuals with chronic diseases and comorbidities, as morbid obesity. This relationship is bidirectional, as some systemic conditions may exacerbate oral symptoms of pain and discomfort, while oral problems may interfere in eating and masticatory behavior (23). The multivariate analysis applied in the present study identified three clusters, named “Young,” “Diabetic individuals,” and “Poor periodontal health,” with unique characteristics and special needs that should be recognized to better assist this condition. A higher number of females composed this convenience sample, probably because de frequency of obesity is higher in females and about 80% of patients who undergo gastroplasty surgery in Brazil are women (24).

Cluster 1 labeled “Young” included younger participants with higher BMI, who reported to use distractors while eating, have a lower number of meals per day and consumed sweetened drinks and processed food the day before examination. In the last years, the dietary patterns have been characterized by an increase in the consumption of high energy density foods, in which those rich in fibers have been replaced by products rich in fats and sugars, with a high level of processing and very low nutritional quality (25). These ultra-processed foods contain little or no whole food and are ready or almost ready for consumption and, therefore, easily accessible and convenient especially for the youth. They are combined with the use of additives, to make them durable and hyper-palatable, being consumed in a higher frequency in lower-middle- and upper-middle-income countries over the years (26). The consumption of this type of food has been shown to be a risk factor for obesity in adolescents and adults (27, 28) and the literature shows that individuals with obesity at a young age tend to remain with this condition throughout their lives (29).

The findings also corroborate a previous study which also noted that distractors, such as reading a printed text or using a smartphone, increase the total calories consumed compared to meals without distractors (30). The same is observed when watching TV (31, 32), and the reasons for this harmful effect seem to be related to distraction and satiety impairment (30, 33). Moreover, it was recently reported that children used to watch TV during meals have lower preferences for vegetables, comparatively to the ones that do not have this distractor during meals (34), what may be also a cause of higher energy intake. Palatable foods, rich in fats and sugars, usually have softer consistency and low content of fibers, preventing food to stay longer inside the mouth to be chewed and, again, impairing satiety (35). Cluster 1 showed the lower demand for oral rehabilitation, probably because it was composed by younger individuals; even so, dietary and nutritional counseling are useful for this groups to improve the quality of what is being ingested, thus preventing negative consequences for oral and general health in the future.

Cluster 2, which included diabetic individuals (whose 73% were insulin users), also showed a great need for nutrition counseling and oral health treatment because of the higher serum glucose levels, loss of teeth, and dry mouth symptoms. The literature shows some evidence on the relationship between diabetes and oral health status, such as the higher risk of severe periodontal insertion losses that may lead to tooth loss; also, diabetic individuals may also show hyposalivation, which can be associated with the xerostomia symptoms reported. It is interesting to note that no statistically significant decreases in the amount of saliva collected was observed; however, saliva collection occurred under stimulation and it is possible that at rest these individuals present lower salivary flow rates. In addition, changes in the oral microbiota, healing difficulties, abscesses and hyperplasia associated with the pathophysiology of diseases or their drug treatments were mentioned (36). Diabetes probably influences periodontal disease because of vascular abnormalities, neutrophils dysfunction, abnormalities in collagen synthesis and genetic factors predisposition (37) and the severe periodontal insertion losses increases the possibility of tooth loss. Previous findings showed that diabetic patients with mild to moderate periodontal disease suffer a more negative impact on quality of life than healthy ones or those with only gingivitis (38, 39). The present results corroborate a previous one (40) which concluded that tooth loss impacts on quality of life independently of the instrument used to measure quality of life or the social context and, in general, studies have shown that the absolute number of teeth as well as their relative position in the mouth are associated with impairments on the oral health-related quality of life (40, 41). According to the results gathered by OHIP-14, it seems logical that the interruption of meals, as well as the exclusion of certain foods from the diet are related to the reduced number of teeth. In the social sphere, the fewer number of healthy teeth is associated with absenteeism at work and to the feeling of social disadvantage (36). The loss of natural teeth significantly reduces the masticatory performance and, thus, acts as a significant barrier in relation to the food choice, and edentulism or partial edentulism are important risk factors for malnutrition (23). The inability to chew or properly grind foods encourage the exclusion of high-fiber foods and favors the consumption of overcooked food (42) and foods with softer consistency, thus potentially affecting the glycemic control.

There are some possible causes for qualitative and quantitative changes in saliva secretion in diabetic patients, as well as the feeling of dry mouth and the reported difficulties in swallowing certain foods and taste changes. Glycosuria, caused by mild hyperglycemia, results in fluid loss and dehydration of the body and can lead to decreased salivary secretion. Also, structural pathologies of complex etiologies can occur in the salivary glands, decreasing their saliva production (43–45). Combined, xerostomia complaints and tooth loss have a great impact on masticatory function and nutrition and, for this reason, it is of utmost important that these patients receive proper nutritional and dietary guidance and dental rehabilitation (46).

Finally, Cluster 3 (“Poor periodontal health”) showed the poorer oral health status, with the most severe periodontal disease, higher number of decayed and filled teeth and lower means of salivary flow, pH, and buffer capacity, and which mostly needs advice on oral health and dental treatment. Periodontitis can negatively affect inflammatory pathways, also affecting the systemic health, which may increase the risk of other diseases such as cardio-metabolic disorders (47). Individuals with obesity appear to be at higher risk for the development of periodontitis as both share a low-grade inflammatory state, and a positive association between them was found in different populations (48). However, the literature has not confirmed a cause and effect relationship between these conditions until present (49).

It is possible that excess weight is a determinant of hyposalivation (50), which is defined as an objective measure of abnormal reduction in salivary flow. Considering the stimulated saliva, rates below 0.7 ml/min are a signal of reduced flow (51, 52). The mean stimulated salivary flow found in this cluster was 0.7 mL/min, that is, some of the individuals presented low saliva flow rate. When it comes to obesity, the decrease in salivary flow can be caused by different mechanisms: the use of drugs for the treatment of comorbidities (hypoglycemic agents, antihypertensive drugs, and antidepressants) that can induce hyposalivation (53); other hypothesis is the fact that pro-inflammatory cytokines derived from adipocytes and macrophages accumulated in adipose tissue can negatively affect the function of the salivary glands due to mild chronic inflammation (54). In the present study, the commonly used drugs reported by the participants were antihypertensives, antidiabetic, antidepressants, and sedatives, and, according to Villa et al. (52), these are the main drugs reported as causing xerostomia/hyposalivation. In addition, the use of multiple drugs (polypharmacy) can promote pharmacodynamic effects and pharmacokinetic drug interactions, increasing the xerogenic potential of drugs (52).

In individuals with excess weight, the decreased salivary flow, pH, and buffer capacity may represent a major risk of oral health problems due to homeostatic imbalances. The impairment in salivary buffer capacity may lead to a reduced protection of the teeth, which are more susceptible to the development of dental caries and halitosis (1). Although the salivary pH found in this sample of individuals within the normal range of 6.8–7.5 (55, 56), the mean pH found in the analysis of buffer capacity may be considered low (57). Considering these findings, a multidisciplinary guidance is a paramount for individuals with morbid obesity to improve their oral and general health conditions.

This is the first study that includes a large sample of individuals with morbid obesity and has many implications for practice and further research. The study pointed out that even when examining a group of individuals with similar socioeconomic level and anthropometric status, this sample of individuals with morbid obesity showed heterogeneous groups (clusters) with different oral and systemic health characteristics and needs. Many of the causes of unsuccessful bariatric surgery are due to poor dietary behavior and compulsions to eat palatable foods that were present before surgery, hence the importance of recognizing the different profiles and planning the target treatments. Indeed, the cross-sectional nature of the findings and the use of a brief dietary questionnaire prevent cause-and-effect conclusions, representing the major limitation of the study, and future prospective studies should explore how diabetes associated to morbid obesity, in addition to insulin and hypoglycemic drugs intake, are related to hyposalivation, xerostomia and nutritional impairments.

## Conclusion

Three clusters of individuals were identified, named “Young” (Cluster 1), “Diabetic individual” (Cluster 2), and “Poor periodontal health” (Cluster 3). Cluster 1 and 2 were the groups that showed higher demand for nutritional and dietetic counseling, because of the poorer eating behavior and higher serum glucose levels, respectively. On the other hand, Cluster 2 and 3 showed the higher demand for oral rehabilitation and dental treatment because of the loss of teeth and worse periodontal condition showed, respectively, besides the need for dietetic counseling. As oral and nutritional health-related aspects may vary given the multifactorial nature of these aspects, the identification of patterns and special needs is of clinical importance to adjust or change unhealthy habits and better assist this condition.

## Data Availability Statement

The raw data supporting the conclusions of this article will be made available by the authors, without undue reservation.

## Ethics Statement

The studies involving human participants were reviewed and approved by Ethical Research Committee of the Federal University of São Paulo (Protocol No. 1201/2017). The patients/participants provided their written informed consent to participate in this study.

## Author Contributions

MM, MG, EL, and PC participated in the study design and conception of the study. MM, SC-J, and EP participated in data and sample collections and data curation. PC and IR supervised the data and sample collection. PC performed the statistical analysis. MM, MG, EL, and PC wrote the manuscript. All authors reviewed and approved the final version of the manuscript.

## Conflict of Interest

The authors declare that the research was conducted in the absence of any commercial or financial relationships that could be construed as a potential conflict of interest.
